# A Case of Transfusion Error in a Trauma Patient With Subsequent Root Cause Analysis Leading to Institutional Change

**DOI:** 10.1177/2324709616647746

**Published:** 2016-05-05

**Authors:** Sean Patrick Clifford, Paul Brian Mick, Brian Matthew Derhake

**Affiliations:** 1 University of Louisville Hospital, Louisville, KY, USA

**Keywords:** transfusion error, massive transfusion protocol, root cause analysis, trauma resuscitation

## Abstract

A 28-year-old man presented emergently to the operating room following a gun-shot injury to his right groin. Our hospital’s Massive Transfusion Protocol was initiated as the patient entered the operating room actively hemorrhaging and severely hypotensive. During the aggressive resuscitation efforts, the patient was inadvertently transfused 2 units of packed red blood cells intended for another patient due to a series of errors. Fortunately, the incorrect product was compatible, and the patient recovered from his near-fatal injuries. Root cause analysis was used to review the transfusion error and develop an action plan to help prevent future occurrences.

## Introduction

Transfusion of the correct blood product is a complex, multistep process. Numerous individuals are involved in the process including physicians, nurses, and laboratory and transport personnel.^1-5^ There are several crucial points where mistakes can occur, such as wristband application, sample collection and testing, selection and labeling of product, identification of the product recipient, and the pretransfusion checking process.^2,6-8^ Two large studies from New York State estimated the risk of erroneous administration of blood product at 1 per 12, 000 to 19, 000 units (U) transfused.^3,6^ The risk of fatal acute hemolytic transfusion reaction due to error has been estimated at 1 per 600, 000 to 800, 000 U transfused.^6,9^

Providing error-free delivery of blood products is a priority in enhancing overall health care safety.^10^ The standard method of error prevention in transfusion medicine has been a 2-person confirmation of both the unit of blood and the patient identification (ID) band. However, several studies have suggested that automation and computerization are major system changes that can potentially prevent clerical errors. Advanced technological systems are gaining greater acceptance in transfusion medicine for this purpose.^4,5,11,12^ Noted advances include the use of computerized barcode-based coupling of the patient ID band with blood product labels as well as radiofrequency identification technology (RFID), which utilizes radiofrequency electromagnetic tagging of blood product linked to RFID-enabled patient ID bracelets.^5,13^ The placement of locking devices over the blood product ports with unique patient and product codes, such as the Bloodloc system, is another measure some institutions are using to reduce human error.^4,5^ Although these systems have shown promise in reducing risk, errors have been reported with each, and there remains significant room for improvement.^14^

We describe the case of a gun-shot victim who was rapidly hemorrhaging from a wound to the femoral blood vessels. During the frenzied resuscitation, the patient was mistakenly transfused product allocated for a patient in an adjacent operating room (OR). The following series of errors led to the incorrect transfusion: the patient care assistant (PCA) broke blood transfer policy by carrying product for 2 separate patients concurrently; the PCA inadvertently left the wrong cooler in the trauma OR; an anesthesia provider did not perform the product check before transfusing. Emergency situations that create an environment of increased transfusion risk include those in which patients are hemodynamically unstable, care providers are distracted, high volumes of product are transfused, and safety policies are omitted in an attempt to expedite care.^3,5,6^ After thorough review and process assessment, our institution developed additional procedures to help prevent transfusion errors in the trauma OR.

## Case Description

A 28-year-old white male with unknown medical history presented to the emergency room after suffering a gun-shot wound to the right groin. Initial vital signs demonstrated a heart rate of 153 beats per minute, oxygen saturation 93%, and a thready pulse with an inability to detect a noninvasive blood pressure. The patient was confused and combative, and pertinent physical exam revealed an actively bleeding wound to the right inguinal region. Central venous access was obtained, Massive Transfusion Protocol (MTP) initiated, and 2 units of un-cross-matched, O-negative blood were hung as the patient was rushed to the Level 1 trauma OR. Lab work had not been obtained prior to transport.

The patient was intubated in the OR without issue and left radial arterial access was obtained. Initial arterial blood gas analysis demonstrated pH 6.622, paO_2_ 424.6 mm Hg, paCO_2_ 57.2 mm Hg, HCO_3_ 6.6 mmol/L, base excess −29.3 mEq/L on 100% FiO_2_, and a point of care hemoglobin 12.1 g/dL. Blood was sent to the laboratory for complete analysis and typing, but those results, as well as cross-matched blood products, were unavailable during the initial resuscitation. When lab results returned, the patient’s coagulation panel demonstrated prothrombin time (PT) 22.4 seconds, partial thromboplastin time (PTT) 49.6 seconds, international normalized ratio (INR) 2.0, and fibrinogen 124 mg/dL. The patient’s blood type was later determined as O-positive.

During sterile preparation of the patient’s abdomen and groin, pulseless electrical activity was detected. Cardiopulmonary resuscitation was initiated, and the surgeons performed a thoracotomy and began internal cardiac massage. On return of spontaneous circulation, the surgeons compressed a completely flaccid aorta while the anesthesia team performed aggressive resuscitation through a rapid infusion system attached to the central line as well as blood warmer systems attached to 2 peripheral intravenous catheters. It is estimated that at least 6 anesthesia personnel participated in the transfusion process in an uncoordinated fashion as the patient demonstrated hypovolemic shock and continued to hemorrhage. Successive MTP coolers of un-cross-matched product were brought into the room by PCAs as fast as the product became available. Laparotomy revealed a bloodless, uncompromised peritoneal cavity. Right groin dissection revealed injuries to the right common femoral artery and vein.

In an adjacent operating room, packed red blood cells (PRBCs) were concurrently ordered for a patient undergoing a spinal fusion, but the product never arrived to the intended location. Investigation of the paperwork in the trauma OR revealed that blood designated for the spinal fusion patient was incorrectly transfused into the trauma patient. Review of events revealed that the PCAs were making numerous trips between the blood bank and the trauma OR secondary to the extensive transfusion requirements. When PRBCs were ordered for the adjacent OR, the blood bank issued products to separate PCAs. However, in an attempt to save time, a hallway exchange occurred, and a PCA began carrying blood products for both patients. While delivering platelets to the trauma room, the PCA mistakenly placed a cooler containing 2 units of O-negative PRBCs intended for the spine patient on the trauma OR floor. In the haste to transfuse the exsanguinating patient, an anesthesia provider grabbed the incorrect PRBC units, assuming they were the O-negative, un-cross-matched, trauma units and transfused without inspecting the paperwork or patient ID band. The incorrect units were transfused through the rapid infuser in a span of less than 2 minutes. When the error was discovered a few minutes later, paperwork analysis demonstrated that the incorrect units were among the last transfused in the resuscitation effort. The blood bank was alerted, and specimens were sent for reanalysis. Fortunately, the transfused blood was compatible, and the patient suffered no secondary morbidity.

The damaged femoral vessels were repaired with an interposition graft, and thrombectomies were performed to the right superficial femoral and femoral profunda arteries. Right calf fasciotomies were performed to prevent compartment syndrome. Nearly an hour after the resuscitation efforts were initiated, the patient achieved hemodynamic stability. Arterial blood gas analysis demonstrated pH 7.40 and base excess −0.1 mEq/L. Intraoperative transfusion included 16 U PRBCs, 11 U fresh frozen plasma, 12 U platelets, and 20 U cryoprecipitate. The patient also received tranexamic acid 1000 mg. Laboratory results immediately following surgery demonstrated hemoglobin 10.7 g/dL, PT 16.3 seconds, PTT 32.6 seconds, INR 1.3, and fibrinogen 184 mg/dL.

At the completion of surgery, the patient was taken intubated to the postoperative care unit. He was subsequently transferred to the intensive care unit neurologically intact and extubated 2 days later. Closure of right lower extremity fasciotomies occurred on hospital day 7. Except for pain management challenges, the remainder of the hospitalization was uneventful, and he was discharged home on hospital day 15.

Following this incident, our institution performed a root cause analysis of the transfusion error with key administrators, blood bank representatives, and the anesthesiologist and nursing personnel involved. Several sources of potential error became apparent. First of all, the training of the PCAs was deemed deficient with regard to blood product transport policy. Second, transfusion protocol at our institution requires a 2-person check of blood product and paperwork with patient wristband verification; a care provider was sacrificing these safety measures in order to expedite care. Third, the review demonstrated that environmental chaos may have contributed to the error. Too many people were in the room attempting to assist in a disorganized fashion. Finally, the labeling of MTP blood products was considered inadequate, and improved tagging could add an additional safety measure to the process. After thorough review, a multisystem action plan was instituted to address these issues. Table 1 details sources of error and potential solutions reviewed by our institution to decrease transfusion risk in the setting of major trauma.

**Table 1.** Root Cause Analysis and Potential Solutions.

Potential Source of Error	Action to Decrease Risk
Deficient training of transport personnel	PCAs are to receive additional education/training focusing on patient safety concerns and blood bank policy regarding product release and transport
Cutting corners to expedite care	The anesthesiologist is to designate unique *Transfusionist* and *Product Verifier* roles
	The anesthesiologist will guide and supervise resuscitation therapy and ensure that appropriate transfusion protocol is followed for all involved
Environmental chaos/distraction	The anesthesiologist is to remove all nonessential personnel from the OR after assigning appropriate care tasks
	A lone PCA will be assigned to the trauma OR and provide services solely for that room
Inadequate MTP product labeling	The blood bank will implement a unique MTP number for each patient and attach that label to the MTP cooler
	All cooler labels will also include the patient’s ID number and the room number for the designated product

Abbreviations: PCA, patient care assistant; OR, operating room; MTP, Massive Transfusion Protocol.

## Discussion

Transfusion error and near-miss events are occurrences that unfortunately remain all too common, and prevention of these mistakes remains a significant medical concern.^1,2,14^ Although most cases are benign, the administration of blood to other than the intended recipient may have disastrous effects.^3^ Transfusion of incorrect blood products to a patient with subsequent acute hemolytic reaction was the second leading cause of death from transfusion from 2005 to 2009 and one of the most frequently avoidable causes of transfusion-related morbidity.^4,9,15^ In order to reduce the human error component of transfusion medicine, some institutions are moving to automated data capture systems such as computerized barcoding and RFID; however, high costs are an implementation barrier at many institutions.^13^ Locking devices such as Bloodloc have also shown promise in reducing transfusion risk, but no system has been able to completely eliminate human error.^14^

Focus groups analyzing transfusion errors have described environments where the opportunity for error is significantly increased. Situations that demonstrate an elevated risk of error include those involving distracted care providers,high-volume transfusion, stressful work conditions, and lack of familiarity with the patient or transfusion policies.^5^ In crises-oriented settings, the process becomes even more compromised.^3^ Many of the reported transfusion errors have occurred in operating rooms and emergency departments, where situations classified as emergent have led to the perceived need to bypass some of the critical safety checks.^3,6^ The Mayo Clinic reviewed nearly 400 000 transfusions at their institution from 2002 to 2005. There were 6 instances of blood product being transfused to the wrong patient. Fifty percent of those events occurred in the OR with O-negative RBCs transfused to the wrong patient.^8^

Our case demonstrates errors made during a resuscitation attempt on a critically ill trauma patient. The time-sensitive nature of the emergency led to mistakes by transport personnel and safety check omissions by anesthesia providers using a blood verification system that many hospitals are moving away from as safer technologies emerge. Transfusion errors in trauma settings are particularly problematic as signs and symptoms of ABO incompatibility such as fever, tachycardia, hypotension, coagulopathy, or hematuria may be misinterpreted as trauma related. Incorrect product may be rapidly infused in large volumes prior to the error being identified. Trauma patients are at risk of hemodynamic instability, coagulopathy, and renal dysfunction; an acute hemolytic transfusion reaction may exacerbate these conditions and significantly increase the likelihood of morbidity or mortality.

The ability to anticipate and understand the possible sources for error is paramount in avoiding the aforementioned scenario. Root cause analysis is a structured approach that can be used for identifying the underlying causes of adverse events and determining specific system vulnerabilities that may lead to errors.^1,16^ We used this technique to analyze the events surrounding the transfusion incident, and a multidisciplinary action plan was enacted to reduce opportunities for mistakes. While advanced automated transfusion systems are not available at our institution at this time, simple measures were implemented to help decrease risk, including changes in labeling and transport technique, the creation of a more structured trauma OR environment, and additional education and training requirements for those involved in blood product transfer.

In conclusion, transfusion error is an all too common event that can result in disastrous consequences. Situations involving large volumes of transfused product in stressful, chaotic environments can exponentially increase the chance for critical errors. Fortunately for our patient, the transfusion error occurred with compatible blood, and no secondary harm was incurred. Significant events of this nature allow for process-assessment and growth through channels such as root cause analysis. Our institution was able to detect flaws in the system, discuss their underlying causes, and look for corrective measures. We identified numerous opportunities for improvement and have implemented changes to help prevent a catastrophic error in the future.
